# A Theoretical Framework for the Development of Need for Cognition in Childhood and Adolescence

**DOI:** 10.3390/jintelligence12100099

**Published:** 2024-10-07

**Authors:** Evelien Aerts, Jeroen Lavrijsen, Franzis Preckel, Karine Verschueren

**Affiliations:** 1 School Psychology and Development in Context, KU Leuven, 3000 Leuven, Belgium; jeroen.lavrijsen@kuleuven.be (J.L.); karine.verschueren@kuleuven.be (K.V.); 2 Department of Mathematics, KU Leuven, 3000 Leuven, Belgium; 3 Department of Psychology, Trier University, D-54286 Trier, Germany; preckel@uni-trier.de

**Keywords:** need for cognition, developmental model, childhood and adolescence, Cognitive adaptation trait theory (CATT)

## Abstract

Extensive research has highlighted the importance of Need for Cognition (NFC) in various contexts, but our understanding of its development remains limited. In particular, the current psychological literature is relatively silent regarding the factors influencing NFC development. We aim to address this gap by proposing a developmental model of NFC based on the principles of the Cognitive Adaptation Trait Theory (CATT). Through a comprehensive review of the current literature, we elucidate the potential key components contributing to the development of NFC in childhood and adolescence. Additionally, we outline several potential strategies to foster NFC development based on the key components of the model. The model aims to provide a starting point for future research on possible mechanisms underlying the development of NFC. Moving forward, future research should empirically test these hypotheses in real-world settings to enhance our understanding of NFC development and validate the suggested fostering strategies on their effectiveness.

## 1. Introduction

In recent decades, Need for Cognition (NFC) has emerged as a significant personality construct that shapes how individuals engage in cognitive activities across various contexts (e.g., Cacioppo et al. 1996). Although extensive research has investigated its role as a predictor of important outcomes (e.g., Liu and Nesbit 2023; Zerna et al. 2023), there remains a notable gap in understanding its developmental aspects (e.g., Jebb et al. 2016). Empirical studies on the developmental trajectory of NFC are currently limited, and research into the factors that influence its development is even scarcer. This highlights an urgent need for further exploration in these crucial areas. This paper serves as a starting point in this line of research by proposing a developmental model of NFC, grounded in a comprehensive review of the existing literature, and by presenting strategies based on this model that future research can utilize to better understand and foster the development of NFC.

### 1.1. Need for Cognition

The notion of NFC was first introduced by Cohen et al. (1955) and initially referred to an individual’s need to structure relevant situations and make the world understandable in integrated, meaningful ways. They depicted NFC as a need, suggesting that the absence of satisfaction could lead to feelings of tension and anxiety, prompting active behaviors to organize the situation and enhance understanding. Building upon this foundation, Cacioppo and Petty (1982) diverged from this drive-reduction framework, shaping the current understanding of NFC. Their focus shifted towards identifying individual differences in the self-reward potential of exerting cognitive activity, and they defined NFC as an individual’s “tendency to engage in and enjoy thinking” (Cacioppo and Petty 1982, p. 116).

In accordance with Cacioppo and Petty’s definition of NFC, a multitude of studies have demonstrated that NFC accounts for systematic variation in individuals’ engagement in cognitively demanding activities. For example, individuals with higher levels of NFC tend to favor complex over simple tasks or activities (e.g., See et al. 2009; Therriault et al. 2014; Zerna et al. 2023), actively search for new information (e.g., Cacioppo et al. 1996; Fortier and Burkell 2014; Petty et al. 2009), and invest more cognitive resources during information-processing than individuals with lower levels of NFC. Furthermore, these NFC-specific behavioral tendencies are all behaviors that are assumed to play an important role in educational and vocational settings (Liu and Nesbit 2023; Von Stumm and Ackerman 2013). In line with this assumption, studies have consistently reported positive associations between NFC and academic performance across primary, secondary, and tertiary educational levels (for a meta-analysis, see Liu and Nesbit 2023) as well as positive correlations between NFC and job performance (e.g., Abdollahi and Rezaee 2014; Sojka and Deeter-Schmelz 2008).

### 1.2. Nomological Network

Since NFC was introduced as a cognitive motivation to engage in and enjoy thinking (Cacioppo and Petty 1982; Cacioppo et al. 1996), it might not be surprising that NFC has been a subject of frequent investigation alongside other motivational factors. Empirical research has shown that NFC exhibits small to moderate positive correlations with academic intrinsic motivation (e.g., Gottfried et al. 2017; Kramer et al. 2021), interests (e.g., Keller et al. 2019; Preckel 2014), academic self-concept (e.g., Keller et al. 2019; Luong et al. 2017), self-efficacy (e.g., Ajadi 2022; Naderi et al. 2018), and mastery (approach) goal orientations (e.g., Meier et al. 2014; Preckel 2014). Moreover, studies have indicated that NFC is linked to the level of intellectual effort an individual commonly puts forth in everyday situations (e.g., Cacioppo et al. 1996). However, NFC is not the only psychosocial factor influencing how individuals typically utilize and invest their intellectual abilities. In terms of conceptualization, NFC fits into the realm of the intellectual investment traits, “a category encompassing various characteristics that influence how, when, and where individuals apply and invest their intelligence” (Von Stumm and Ackerman 2013, p. 842). Over the years, a considerable number of intellectual investment traits have been proposed (e.g., Von Stumm and Ackerman 2013), some of which have been shown to be closely related to NFC. Specifically, Typical Intellectual Engagement (TIE; Goff and Ackerman 1992), Epistemic Curiosity (EC; Berlyne 1978), and Openness to Ideas (OI; Costa and McCrae 1992) seem to exhibit substantial conceptual overlap with NFC, resulting in strong positive correlations between NFC and these traits (e.g., Mussel 2010, 2013; Powell et al. 2016; Woo et al. 2007). While some studies have shown that multiple factor models provide the best fit to the data when comparing NFC, TIE, EC, and OI (Fleischhauer et al. 2009; Mussel 2013; Powell et al. 2016), providing evidence for these related traits being different constructs, other studies have found one-factor models to provide the best fit (Mussel 2013; Powell and Nettelbeck 2014). Consequently, some researchers have raised concerns about NFC’s discriminant validity in relation to these factors (Mussel 2010; Powell et al. 2016; Woo et al. 2007), encouraging efforts to integrate these investment traits into comprehensive frameworks, such as the Intellect framework (Mussel 2013). However, the conceptual similarities between NFC, TIE, EC, and OI could also be highlighted. Table A1 (see Appendix A) summarizes the aforementioned investment traits, delineating their conceptual differences and similarities concerning NFC, thus underscoring the importance of recognizing them as distinct constructs.

### 1.3. Development of Need for Cognition

Although NFC was initially introduced as a stable interindividual difference (i.e., a personality trait), Cacioppo et al. (1996) underscored its malleability, suggesting that NFC is not fixed and can be cultivated or changed. Despite this, longitudinal research on the development of NFC is limited. We only encountered four longitudinal studies that investigated the developmental trajectory of NFC. A first study was conducted by Bruinsma and Crutzen (2018), who examined changes in NFC within a six-year timeframe among individuals aged 16 to 95. The participants’ NFC scores were assessed five times: between May and August 2008 (T1), May and June 2009 (T2), May and June 2011 (T3), May and June 2013 (T4), and November and December 2014 (T5). Their findings indicated that significant mean-level changes did occur over time, but that these changes in NFC levels differed in magnitude and direction between the different age groups: whereas the average NFC levels of younger participants (i.e., 16 to 25 years old) increased (Δ_NFC≤24_ = 0.241), the NFC levels of middle-aged (i.e., 25 to 49 years old) and older respondents (i.e., 50 to 95 years old) decreased (Δ_NFC25–49_ = −0.059 and Δ_NFC≥50_ = −0.098). In line with these observed mean-level changes, the middle-aged respondents descriptively had a higher autoregressive correlation (*ρ* = 0.189, *p* < .001) compared to the younger respondents (*ρ* = 0.119, *p* = 0.037) and older participants (*ρ* = 0.106, *p* < 0.001), suggesting that NFC scores at one point in time are a better predictor of subsequent scores in middle-aged respondents compared to the other age groups. Bruinsma and Crutzen (2018) also considered the interindividual differences in change over time, which were found to be significant. Interestingly, their results showed that these interindividual differences were more pronounced in the youngest age group (*σ*_≤24_ = 0.005, *p* < .001) compared to the older respondents (*σ*_25–49_ = 0.002, *p* < .001 and *σ*_≥50_ = 0.001, *p* = .003). Bergold et al. (2022) examined NFC from mid-to-late adolescence over a period of three years with four times of measurement: at the onset of the 11th grade (T1), six months later at the beginning of the second 11th grade term (T2), at the beginning of the second term in 12th grade (T3), and at the start of the second term in 13th grade (T4). Their analyses not only revealed a significant positive increase in mean NFC scores throughout this developmental period (*μ*_slope_ = 0.007, *p* < .001) but also identified significant interindividual differences in these growth trajectories of NFC (*σ*_slope_ < 0.001, *p* = .042). Additionally, they found the rank-order stability of NFC to be *r* = 0.59, which indicates a medium stability of a participants’ rank within the study sample over time. A third study, by Bergold and Steinmayr (2023), investigated reciprocal relationships among several investment traits, including NFC, and intellectual abilities over a period of one year in German elementary children aged 8 to 9. The NFC scores of these children were measured at the beginning of the study (T1) and approximately one year later (T2). Their findings revealed a slight, albeit non-significant, decrease in NFC (Δ_NFC_ = −0.07), with the rank-order stability of NFC being *r* = 0.56 over this one-year interval. However, the authors also emphasized that there was significant interindividual variation in the change of NFC levels among the children (*σ*_Δ_ = 0.19, *p* ≤ .001). Lastly, Preckel (2014) focused on validating a 19-item NFC-scale for children in a sample of German students between 10- and 12-years-old. Within this study, the NFC levels of the same group of students were measured at the beginning (T1) and the end (T2) of the 5th grade, and at the end of the 6th grade (T3). Across these three measurement points, there was a significant decline in mean NFC scores. Preckel (2014) did not consider the rank-order stability of or the interindividual variation in this decline in NFC. In addition to these longitudinal studies, cross-sectional research can also provide valuable information about the development of NFC. For instance, Luong et al. (2017) measured NFC scores across three different age groups—third grade, sixth grade, and ninth grade—and found that mean NFC scores significantly decreased from third to ninth grade. Similarly, Keller et al. (2019) reported a significant decline in NFC scores from first to fourth grade and further demonstrated that the NFC scores of ninth-grade students were significantly lower than those of seventh-grade students.

Taking together the results from the available longitudinal and cross-sectional studies, a general decline in NFC from childhood to mid-adolescence seems likely. Nonetheless, research involving older samples suggests a general increase in NFC in late adolescence and early adulthood. These trends align with those found for Openness to Experience, the Big Five trait most closely related to NFC (Fleischhauer et al. 2009), which generally decreases across childhood and mid-adolescence and increases in late adolescence and young adulthood (Atherton et al. 2020). This U-shaped trajectory has been refered to as the disruption hypothesis, which posits that individuals undergo declines in socially desirable traits throughout adolescence, likely influenced by various biological, psychological, and social changes during this developmental phase (Soto and Tackett 2015). In late adolescence and early adulthood, however, young people again resume mean-level increases in these socially desirable traits, including Openness to Experience (Atherton et al. 2020).

Besides the mean-level change of NFC, which reflects changes at the group level, it is also important to look at the individual differences within the developmental trajectories of NFC. In this context, two of the aforementioned longitudinal studies provided data on the rank-order stability of NFC, suggesting medium rank-order stability in both childhood and adolescence. Similar findings have been demonstrated in research on the rank-order stability of Openness to Experience in youth. However, the two studies only measured the rank-order stability of NFC over a short time period (i.e., one year and three years), giving us no information about the rank-order stability of NFC over longer periods (e.g., from childhood to young adulthood), as it has been shown that the rank-order stability of personality traits tends to decrease when measured over longer periods of time (Roberts and Delvecchio 2000). Furthermore, the studies discussed above demonstrated significant interindividual variation in NFC trajectories among both childhood and adolescent samples. These results suggest substantial differences among individuals in how their NFC evolves over time, prompting the question: which factors influence NFC development?

### 1.4. Current Study

Based on the studies discussed above, we can conclude that NFC does indeed change across the lifespan, as indicated by the observed mean-level changes. Moreover, the significant interindividual differences found in these studies suggest that certain factors may influence this development for better or for worse. However, this raises the question as to which factors influence the changes in this enjoyment of thinking. The literature on personality development suggests several potential influences on trait development, including genetics (i.e., inherited characteristics), the active selection or evocation of environments (i.e., selecting or evoking environments based on genetic preferences, motivations, and traits), and various environmental factors (Briley and Tucker-Drob 2014; Giannoukou 2024). Together, these different factors interact in complex ways to shape NFC over time.

Despite the widespread acknowledgement of the importance of NFC across various contexts, the current literature lacks a substantial exploration of the factors shaping NFC development. The majority of research has centered on the predictive capacity of NFC itself, while the consideration of its determinants remains limited. Although some researchers have made theoretical propositions on what could influence NFC development, a comprehensive theory regarding the antecedents of NFC is still lacking. However, given the (initial) potential decline in NFC throughout the school years and its association with numerous positive outcomes, fostering NFC development seems crucial. This paper aims to address this gap by using an emergent trait theory—the Cognitive Adaptive Trait Theory (CATT; Matthews 2017)—to suggest a developmental model of NFC. We will outline the key components of the CATT and, drawing from the current literature on NFC, elucidate how they may interact and lead to the investment of cognitive effort. Furthermore, we will suggest possible strategies to foster NFC development and expound on their mechanisms using the suggested model. Beyond providing a framework for understanding NFC’s origins, this model will also provide avenues for future research to test its predictions, which will further contribute to our understanding of NFC development.

## 2. Towards a Developmental Model of NFC

### 2.1. Cognitive Adaptive Trait Theory (CATT)

**Figure 1:**
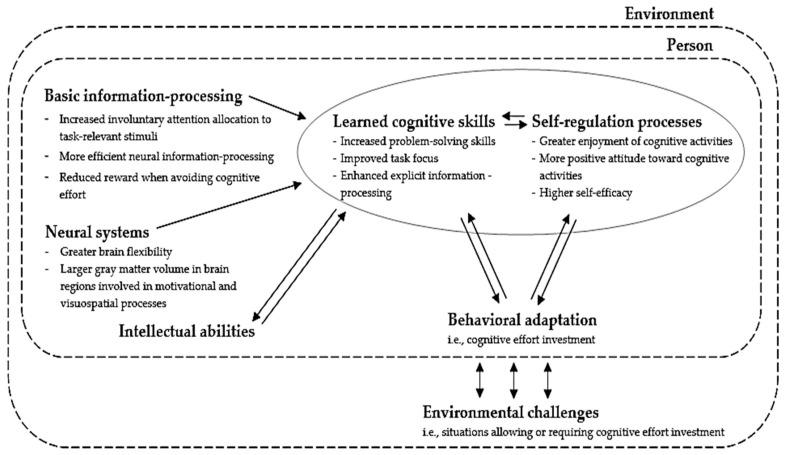
Developmental model of Need for Cognition based on the CATT.

The developmental model of NFC, based on the CATT, is depicted in Figure 1. The core idea of the CATT is that trait diversity manifests through behavioral adaptations to environmental challenges, reflecting a range of strategies individuals use to handle these challenges (Matthews 2008). Within this framework, the dimension “environmental challenges” is used rather broadly, covering all situations and contexts that allow or require individuals to respond with adaptive strategies. For instance, navigating challenges related to interpersonal relationships is a key aspect of the trait Agreeableness (e.g., McCrae and John 1992). In environments that value social harmony, establishing and maintaining positive relationships may come easily. However, when faced with social conflict, the challenge becomes more complex. In such cases, the ability to balance the need for harmony with the necessity of standing up for oneself or others is crucial. This illustrates how traits like Agreeableness are shaped as adaptations to environmental challenges.

Furthermore, the CATT proposes that each trait comprises multiple dimensions that influence and reinforce each other over time. Traits are not fixed characteristics like blood type; instead, they are understood as distributed across various processes, unified by their adaptive functions. Two key dimensions of the CATT are “learned social-cognitive skills” and “self-regulation” (Matthews 2008, 2017). Learned social-cognitive skills include all acquired skills that that help individuals to adaptively manage the aforementioned environmental challenges, such as stress management or effective conflict resolution strategies (Matthews 2020). Self-regulation, on the other hand, involves mechanisms that oversee the application and effectiveness of these skills. Particularly in challenging environments, individuals must monitor how well their skills are working, process feedback, set realistic goals, and determine if additional effort or adjustments are needed to overcome the lack of skills. These regulatory processes are also closely tied to emotional responses, which can be positive (e.g., satisfaction from achieving a goal) or negative (e.g., frustration from a failure). Within the CATT, self-regulation is defined more comprehensively than in the general psychological literature and includes various constructs from personality research that potentially mediates trait expression, such as self-esteem, self-efficacy, affective states, and appraisal (Matthews 2017). To avoid confusion with the typical use of “self-regulation” in the literature, the term “self-regulation processes” will be used from now on to refer to this category of psychological mechanisms. This terminology does slightly differ from the original CATT, but, according to us, better captures the intended meaning of this component of the model.

The acquisition of these social-cognitive skills and self-regulatory processes are supported by a range of neural (e.g., gray-matter volume) and basic information-processing mechanisms (e.g., working memory), which are assumed to indirectly influence adaptive behaviors by shaping the skills and self-regulatory processes. Conversely, social-cognitive skills and self-regulatory processes are assumed to impact adaptive behavior more directly. Moreover, these three aspects—behavioral adaptations, social-cognitive skills, and self-regulatory processes—are intertwined in a continuous feedback loop; adaptive behavior can drive the acquisition of cognitive skills or changes in self-regulatory systems, which then reinforces adaptive behavior once again (Matthews 2017). Additionally, social-cognitive skills and self-regulatory processes constantly interact with each other, further strengthening this feedback loop.

For instance, consider individuals with high Agreeableness. Research indicates that such individuals show heightened activity in brain regions associated with empathy (Li et al. 2017), and they tend to process antisocial stimuli more superficially and prosocial stimuli more deeply compared to those with lower levels of Agreeableness (Wilkowski et al. 2006). These underpinnings facilitate the acquisition of several social-cognitive skills and self-regulatory processes commonly linked to higher Agreeableness, such as effective conflict resolution strategies and enhanced social self-efficacy (e.g., Field et al. 2014). In turn, these skills and self-regulatory processes increase the chance of behaviors frequently related with high Agreeableness, such as prosocial behavior and conflict resolution (e.g., Tehrani and Yamini 2020). Moreover, skills and self-regulatory processes also interact and thereby reinforce each other, further enhancing agreeable behaviors over time: individuals high in Agreeableness may use their enhanced resolution skills to better navigate conflicts, leading to more effective resolutions, which in turn boosts their confidence in handling future conflicts and strengthens their overall ability to do so.

### 2.2. Application of the CATT to NFC Development

The CATT framework can also be applied to the development of NFC, which was introduced as a personality trait (Cacioppo and Petty 1982). Since traits are defined as strategies for adapting to key environmental challenges within the CATT, the adaptive challenges in the case of NFC can be regarded as any situation or activity allowing or demanding the investment of cognitive effort. While social skills may play a less central role in NFC development, the current literature does suggest an influence of several cognitive skills and self-regulatory processes on the emergence of cognitive effort investment (i.e., the behavioral adaptation of NFC). Empirical research has demonstrated significant positive relations between NFC and various cognitive skills and self-regulatory processes, suggesting a potential impact on whether an individual regularly exerts cognitive effort or not. Additionally, some positive associations between NFC and neural and basic information-processing elements have been found.

As previously noted, traits are not fixed characteristics but are distributed across multiple processes (depicted in Figure 1), gaining coherence through their adaptive functions. NFC should not be viewed as a distinct trait with these specific correlates. Instead, NFC, as measured by standard assessments, relates to these variables, suggesting that it is part of a complex but integrated set of processes designed to adapt to the key environmental challenge of cognitively demanding situations. Consequently, while skills may not be directly tapped by typical NFC questionnaires, which rather focus on the self-regulatory processes and behavioral adaptations of NFC, they are regarded as inextricably intertwined with self-regulatory and behavioral processes comprised by the overall trait concept. In the following section, we will delve into these elements and highlight their dynamic interplay, since we expect that this interplay constitutes the primary driving force behind positive NFC development. Table A2 (see Appendix B) consists of an overview of the aforementioned elements of the developmental model of NFC and its empirical evidence.

### 2.3. Neurobiological and Basic Information-Processing Elements

A few studies have tried to uncover the neural and basic information-processing elements contributing to individual differences in NFC levels. In terms of neural correlates, research indicates that NFC is positively associated with larger gray-matter volume in brain regions involved in motivational and visuospatial processes, as well as with greater brain flexibility across various brain areas and networks (He et al. 2019; Tolomeo et al. 2023). Concerning basic information-processing elements, EEG research has linked higher NFC levels to electrocortical indices reflecting increased voluntary and involuntary attention allocation to task-relevant stimuli (Enge et al. 2008; 2011; Strobel et al. 2015). This suggests that individuals with high NFC not only choose to focus their attention on more relevant stimuli when faced with intellectually challenging tasks (i.e., top-down, explicit), but their attention is also automatically drawn to these relevant stimuli (i.e., bottom-up, implicit). Furthermore, individuals with high levels of NFC tend to exert more cognitive effort when confronted with situations possessing high (compared to low) cognitive demand, reflected in specific patterns of theta oscillations, which indicates neuronal efficient information-processing. In contrast, those with lower NFC levels do not exhibit such tailored responses across tasks varying in cognitive demand (Mussel et al. 2016). Lastly, a study relying on self-report has shown that individuals high in NFC experience lower reward when high effort is avoided compared to individuals low in NFC (Gheza et al. 2023). However, this hypothesis warrants further exploration in neurobiological research.

### 2.4. Intellectual Abilities

While the role of intellectual abilities is not explicitly highlighted in the CATT, its significance in the development of NFC cannot be disregarded. From a conceptual standpoint, there are two reasons why NFC should be related to intellectual abilities (Lavrijsen et al. 2023). First, individuals with higher levels of intelligence are generally more likely to excel in cognitive activities, which in turn could lead to a heightened appreciation of and more frequent engagement in cognitive effort (i.e., higher NFC). This assumption is also known as the environmental success hypothesis (Ziegler et al. 2012). Second, frequent engagement in effortful cognitive activities is expected to enhance one’s intellectual abilities. Thus, individuals who are inherently drawn to cognitive activities (i.e., have higher NFC) may be more inclined to develop their intellectual abilities, also known as the environmental enrichment hypothesis (Ziegler et al. 2012). In line with these hypotheses, empirical research has identified positive correlations between NFC and intelligence measurements, which seem to increase from very small in children to moderate in adults, aligning with the positive reciprocal effects between both constructs (e.g., Bergold and Steinmayr 2023; Fleischhauer et al. 2009; Hill et al. 2013; Lavrijsen et al. 2021; Von Stumm and Ackerman 2013). Also, although empirical research usually finds only small positive correlations between NFC and intelligence in children, Ackermann et al. (2022) found that implementing inductive reasoning training within a group of pre- and primary school children not only positively impacted intelligence measures but also had a small positive effect on NFC scores post-training. However, the positive effect of the reasoning training on NFC was not maintained at the follow-up assessment, which was conducted three months after the intervention had concluded. These findings align with the pattern observed in many other intervention studies aimed at enhancing intellectual abilities: while initial improvements are often noted at post-test, these gains frequently diminish over time, also known as the fadeout effect (for a meta-analysis, see Protzko 2015).

Nevertheless, it is crucial to distinguish between NFC and intellectual abilities. While stronger intellectual abilities may positively influence the development of NFC, as suggested by the environmental success hypotheses, it is not a prerequisite for inherently enjoying the exertion of cognitive effort. A number of studies showed that NFC incrementally predicted academic performance, school engagement, and intrinsic motivation above and beyond intellectual abilities (e.g., Lavrijsen et al. 2023; Preckel 2014; Strobel et al. 2019). This underscores the unique role of NFC in intellectual engagement and achievement, highlighting its importance beyond traditional measures of intellectual abilities.

### 2.5. Learned Cognitive Skills

While intellectual abilities pertain to higher-order cognitive functions, such as reasoning and abstract thinking, which have been found hard to change (i.e., fadeout effect; Protzko 2015), cognitive skills refer to acquired skills related to information-processing and learning, which can more easily be enhanced through training, practice, and experience (e.g., Ackerman 1996; Hung et al. 2012; Yang 2012). Despite their differences, more general intellectual abilities and learned cognitive skills are related. Higher levels of intellectual abilities often provide a solid foundation for acquiring and developing cognitive skills (Ackerman 1996; DeYoung et al. 2008). However, the distinction is not always clear-cut. In the context of our model, learned cognitive skills encompass all acquired abilities that help individuals adapt to the environmental challenges of NFC (i.e., situations allowing or requiring effort investment). Below, we discuss several examples, though this is by no means an exhaustive overview of the various cognitive skills that can be developed to handle such challenges.

Throughout the past few decades, NFC has been thoroughly investigated in relation to a wide array of learned cognitive skills. For instance, it has been consistently observed that higher levels of NFC are linked to increased skills to solve problems (Coutinho et al. 2005; Nair and Ramnarayan 2000; Rudolph et al. 2018) and improved task focus (Levin et al. 2000; Li and Browne 2006; Srivastava et al. 2010). Moreover, NFC has been associated with the use of strategies that enhance information-processing and deeper learning, such as structuring or reflective learning (e.g., Cazan and Indreica 2014; Loes and An 2021; Mokhtari et al. 2013). The placement of information-processing skills within the category of learned cognitive skills may lead to some confusion, especially in light of our earlier discussion on basic information-processing. However, a distinction between these categories can be made. While basic information-processing refers to elementary cognitive processes such as perception and attention, information-processing skills encompass more complex skills that help to process information, including tasks such as decoding, language processing, comparing information, and employing control strategies. The idea that information-processing skills play a role in the development of NFC is in line with the elaboration likelihood model suggested by Petty and Cacioppo (1986), who proposed two distinct routes for processing persuasion-related information: the central route and the peripheral route. Those with high levels of NFC are presumed to predominantly engage in information-processing via the central route, indicating deeper scrutiny of available information. Conversely, individuals with low NFC are anticipated to utilize the peripheral route for information-processing, relying more on noticeable cues such as source characteristics, which results in more superficial information-processing. Therefore, the existence of a relationship between NFC and enhanced information-processing skills may not be surprising. Collectively, these learned cognitive skills enable individuals with higher NFC to effectively handle situations that allow or demand significant cognitive effort.

### 2.6. Self-Regulatory Processes

Cacioppo and Petty’s conceptualization of NFC emphasizes that individuals with high NFC typically derive enjoyment from engaging in cognitively challenging activities, which has been empirically validated in self-report studies (e.g., Li and Browne 2006). Additionally, several studies utilizing both self-report and objective measures have shown that people with varying NFC levels differ in their attitude toward and evaluation of the effort involved, with individuals possessing higher levels of NFC evaluating such endeavors more positively (Watts et al. 2017; Weissgerber et al. 2018; Westbrook et al. 2013). Moreover, it has been observed that individuals with higher levels of NFC tend to prefer complex over simple problems, while individuals with lower NFC levels do not exhibit this preference for complex problems and may even try to avoid the exertion of cognitive effort altogether (See et al. 2009; Therriault et al. 2014; Zerna et al. 2023). When individuals engage in activities that require self-regulation, such as a cognitively demanding tasks, they must override certain responses (e.g., distractions, giving up when faced with difficulty). This can be exhausting and may subsequently lead to diminished self-regulation. Interestingly, it has been demonstrated that positive emotions such as enjoyment can counteract the negative effects of these self-regulation demands, and thereby facilitate subsequent self-regulation. For example, Tice et al. (2007) found that after an initial act of self-regulation, participants who were induced with positive emotions through watching a comedy video persisted longer on both solvable and unsolvable puzzles than those who watched a neutral video. Thus, individuals high in NFC, who inherently enjoy engaging in intellectually demanding tasks, may benefit from these positive emotions during such tasks through the enhancement of their ability to sustain self-regulatory efforts over time.

Furthermore, the role of perceived self-efficacy in the development of NFC also warrants consideration, as previously suggested by several researchers (Elias and Loomis 2002; Jebb et al. 2016). Perceived self-efficacy refers to an individual’s belief to master challenges and to carry out the actions required to achieve specific outcomes (e.g., Bandura 1997). Since self-efficacy is not a unitary construct, the self-efficacy beliefs of people may be different across various domains. In the context of the promotion of NFC, academic or learning self-efficacy could play an important role (Elias and Loomis 2002; Jebb et al. 2016). Academic self-efficacy pertains to students’ beliefs that they have the capability to successfully complete academic tasks (e.g., Bandura 1997; Eccles and Wigfield 2002; Elias and Loomis 2002). Students who believe in their capacity to tackle intellectually challenging tasks are more likely to be engaged and persistent when faced with such tasks (e.g., Bandura 2016; Ouweneel et al. 2013; Usher et al. 2019). Additionally, students with higher self-efficacy levels tend to experience positive emotions, such as enjoyment, in learning contexts (e.g., Hayat et al. 2020; Mega et al. 2014; Pekrun et al. 2011). Since both engagement in and enjoyment of effortful cognitive activity are crucial elements in the definition of NFC, it might not be surprising that research has demonstrated that NFC and self-efficacy are positively related (Ajadi 2022; Elias and Loomis 2002; Naderi et al. 2018). Theoretically, it is reasonable to assume that self-efficacy plays a role in the development of NFC, as individuals are more likely to seek out and enjoy cognitive tasks when they believe in their ability to successfully complete them (Elias and Loomis 2002; Jebb et al. 2016; Naderi et al. 2018). However, an empirical validation of this assumption is currently lacking.

### 2.7. Behavioral Adaptation

As previously discussed, the exertion of cognitive effort in response to environmental challenges labeled as “situations that allow or require cognitive effort” can be understood as a behavioral adaptation of NFC. According to Cacioppo et al. (1996), NFC reflects individual differences in the intrinsic motivation to engage in cognitive effortful activities. Research supports this notion, demonstrating a positive association between NFC and both the willingness to exert cognitive effort (e.g., Kramer et al. 2021) and the frequency of actually engaging in situations that allow or require such effort (e.g., Cacioppo et al. 1996; Cazan and Indreica 2014; Therriault et al. 2014). For example, Therriault et al. (2014) demonstrated a positive correlation between NFC and participation in more cognitively demanding leisure activities, as opposed to those requiring less cognitive effort, in university students. Moreover, NFC not only influences how frequently individuals seek out cognitively challenging activities but also their approach once these situations are encountered: NFC has been linked to both observational and self-report measures of persistence and engagement in cognitively demanding tasks (Dickhäuser et al. 2009; Fleischhauer et al. 2015; Lavrijsen et al. 2023).

Higher levels of NFC have also been consistently related to greater academic achievement (for a meta-analysis, see Liu and Nesbit 2023) and job performance (e.g., Abdollahi and Rezaee 2014; Sojka and Deeter-Schmelz 2008). This relationship may be interpreted as a more distal effect of the behavioral adaptation of NFC: individuals with high NFC tend to more frequently seek out and invest greater cognitive effort in their daily activities, which subsequently fosters improved academic and vocational outcomes.

### 2.8. Dynamic Interplay between These Elements

**Figure 2:**
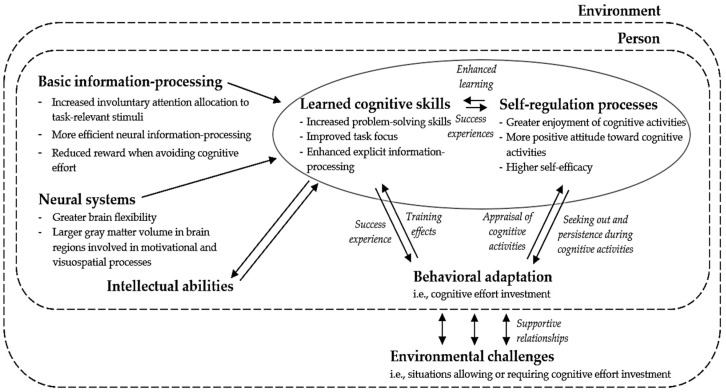
Interactions within the developmental model of Need for Cognition.

Apart from merely exploring the cognitive skills, self-regulatory processes, and behavioral adaptations associated with NFC in isolation, delving into the dynamic interplay among these elements will further deepen our understanding of NFC development. Firstly, learned cognitive skills and self-regulatory processes, illustrated in the inner circle of the model, are thought to directly impact the frequency and manner with which individuals engage in effortful cognitive activities. Secondly, as depicted by the adaptive triangle in the model, the continuous interaction among cognitive skills, self-regulatory processes, and behavioral adaptations creates opportunities for these elements to mutually reinforce one another over time, leading to increased cognitive effort investment. Figure 2 summarizes these interactions. For instance, individuals with good cognitive skills, such as enhanced information-processing and skills to solve problems, are more likely to excel in intellectual challenges, leading to success experiences that cultivate a positive effect in learning situations and strengthen self-efficacy beliefs through positive appraisal (Hendricks 2014; Kleppang et al. 2023; Pekrun 2014). Conversely, individuals who generally derive enjoyment from engaging in cognitive activities and possess confidence in their ability to do so are more inclined to actively seek out, engage in, and persist during such endeavors (e.g., Oriol et al. 2017; Rodríguez-Muñoz et al. 2021; Schunk and Dibenedetto 2021). This increased cognitive investment exposes them to a diverse range of cognitive activities, thereby providing consistent training opportunities that facilitate skill refinement over time. Additionally, research indicates that positive learning-related emotions, such as enjoyment, can boost the learning process: individuals who find pleasure in tackling challenging cognitive tasks tend to exhibit greater learning outcomes compared to those who do not find such tasks enjoyable (Chen et al. 2021; Li et al. 2020; Tan et al. 2021). Lastly, how the exertion of cognitive effort is appraised can also influence the self-regulatory processes of NFC. A positive appraisal of cognitive effort, such as viewing it as enjoyable, motivationally relevant, manageable, and valuable, can lead to increased enjoyment of, self-efficacy toward, and engagement in demanding cognitive activities (Forsblom et al. 2022; Lizzio and Wilson 2013; Schmidt et al. 2010).

## 3. Fostering Need for Cognition

Figure 2 illustrates that learned cognitive skills, self-regulatory processes, and behavioral adaptations associated with NFC exhibit ongoing interaction, influencing each other over time in varying manners. More interestingly, these dynamic associations present avenues through which the environment can positively shape NFC development. According to the CATT model (Matthews 2017), the more changeable components are situated within this adaptive triangle. Although neurobiological systems, basic information-processing elements, and intellectual abilities may enhance the likelihood of developing certain cognitive skills and self-regulatory processes pertinent to positive NFC development, the potential for an environmental influence on these cognitive and self-regulatory skills remains substantial. In contrast, neurobiological systems, basic information-processing elements, and intellectual abilities have been shown to be less prone to lasting change caused by the environment (e.g., Matthews 2017; Protzko 2015). In the subsequent section, we will propose several strategies, derived from the developmental model of NFC, through which the environment can actively cultivate contexts that are possibly supportive for NFC in youth, by impacting the dynamic interplay among cognitive skills, self-regulatory processes, and behavioral adaptation. The proposed strategies will specifically target the enhancement of cognitive skills, self-regulatory processes, and behavioral adaptations. While Figure 2 can aid in understanding how the various strategies are related to the proposed model, Table A3 (see Appendix C) offers a clear overview of these strategies.

### 3.1. Safe Learning Environment as a Prerequisite

While the strategies suggested below certainly are promising, it is likely that the environment must possess several crucial characteristics before these strategies may take effect. One such potential prerequisite for nurturing positive NFC development is the quality of interpersonal relationships, particularly with significant others such as parents or teachers. Attachment theory suggests that close relationships facilitate exploration by offering a safe haven for children to seek comfort during distress (Bretherton 1992). When significant others are perceived as caring and responsive, young individuals tend to experience less anxiety regarding threats or failure during exploration (Heatly and Votruba-Drzal 2019; Ryan and Deci 2013). Consequently, nurturing supportive relationships with caregivers through expressing care, acceptance, and support is likely to shape a child’s readiness to engage in intellectually challenging pursuits, as they feel supported and encouraged to explore, take risks, and seek assistance if needed (Kashdan and Fincham 2012). In line with this assumption, substantial research suggests that students demonstrate heightened effort, engagement, and intrinsic motivation when they perceive their teachers and parents as understanding and caring (e.g., Engels et al. 2021; Fedesco et al. 2019; Weyns et al. 2018). Aligning this possible prerequisite with the proposed model, it can be hypothesized that supportive relationships may help young people view environmental challenges not as threats but as opportunities, thereby increasing their willingness to engage with them. However, to our knowledge, there is currently no research that directly investigates the link between NFC and a safe learning environment. This gap in the literature warrants further investigation to better understand how such environments might influence the development of NFC, potentially offering new insights into effective educational practices.

### 3.2. Optimal Challenge

It has been put forward that an intellectually stimulating environment could potentially enhance the development of NFC (Jebb et al. 2016), which underscores the importance of providing students with opportunities to engage in cognitively challenging activities to enhance their NFC. While there is some support for this proposition in longitudinal studies (Loes and An 2021; Padgett et al. 2010), it is not yet clear what exactly characterizes such a stimulating environment in the context of positive NFC development. The recent literature underscores the importance of optimal challenge, where task demands align with or slightly exceed individual capacities (Lavrijsen et al. 2021; Shernoff et al. 2003). Given that an appropriately challenging environment increases the likelihood of success experiences with intellectual tasks, students in such environments tend to exhibit heightened engagement and have more positive beliefs regarding their competence to tackle such activities (Deci et al. 1996; Renninger and Hidi 2015; Shernoff 2013). However, it is not merely the heightened likelihood of success that brings forth the positive outcomes of optimal challenge. Activities that match or slightly exceed one’s abilities tend to also increase enjoyment and feelings of fulfillment during the activity, as achieving something not immediately evident fosters learning and personal growth (Abuhamdeh and Csikszentmihalyi 2012). Conversely, excessively easy schoolwork, despite a high likelihood of success, can lead to boredom and disengagement (Krannich et al. 2022). Moreover, when individuals feel optimally challenged during a cognitive activity, they are more likely to have a fulfilling subjective experience and to be intrinsically motivated to engage in such activities both presently and in the future (Lavrijsen et al. 2021; Reeve and Deci 1996). Therefore, creating an optimally challenging environment is a promising strategy for enhancing NFC development, as it boosts the likelihood of success experiences and thereby positively influences enjoyment and self-efficacy—key components of the self-regulatory dimension of the model—through positive appraisals of these successful outcomes. In addition, such environments provide the ideal foundation for developing cognitive skills (Csikszentmihalyi et al. 2014), further contributing to positive NFC development. The idea that optimal challenge could be crucial in the context of NFC development is supported by the findings of Loes et al. (2012), who observed that college students perceiving their classes as optimally challenging reported increases in NFC during their first year of college.

Despite these promising findings, it is essential to note that mere exposure to optimal intellectual stimulation might not suffice to foster NFC; it rather serves as a prerequisite for positive NFC development. Various studies have indicated that NFC seems to be largely unaffected by socio-economic background (Colling et al. 2022; Padgett et al. 2010; Preckel and Strobel 2017). Given the strong correlation between socio-economic background and exposure to cognitive activities at home (Bodovski and Farkas 2008), this suggests that the provision of optimal intellectual stimulation may not be sufficient. It could be that students must also learn to appreciate and embrace cognitive challenges. The strategies suggested below could provide valuable insights into this matter.

### 3.3. Appraisal of Cognitive Activities

According to the appraisal theory, an individual’s emotional response to an event is shaped by their evaluation of the event across various appraisal dimensions, such as motivational relevance, goal congruence, coping potential (i.e., self-efficacy), and alignment with internal values (e.g., Lazarus 1991; Scherer and Moors 2019). Consequently, differences in emotional and behavioral reactions to the same event can be attributed to individual differences in how the event is appraised across these dimensions. Based on this theory, it could then be hypothesized that individuals are more likely to voluntarily seek out, engage in, and enjoy cognitively effortful activities when they appraise such tasks as motivationally relevant, manageable, and in alignment with their values and goals. Enhancing the appraisals of cognitively effortful tasks could, therefore, foster NFC development.

#### 3.3.1. Appraisal of Value

Several empirically supported ways to enhance these appraisals exist. For example, it has been shown that providing students with tasks that match their interests, as opposed to boring tasks, leads to increased appraisals of value and enjoyment during these tasks (Hulleman et al. 2008; Patall 2013). Another effective approach to positively alter appraisals of value is through explicit highlighting or appraising of its value. However, it could also be effective if teachers, parents, or other influential figures explicitly emphasize the value of cognitive activities in younger populations (e.g., Acee and Weinstein 2010; Gaspard et al. 2015; Shin et al. 2018). For example, Gaspard et al. (2015) assigned ninth-grade students to either one of two relevance-inducing conditions (writing a text or evaluating value statements from other students) or a control condition (no intervention). While both relevance-inducing tasks increased the utility value (i.e., perceived usefulness of performing a task) reported by the students, only the condition involving the evaluation of value statements increased their attainment value (i.e., the importance attached to doing well) and intrinsic value (i.e., the enjoyment derived from doing a task). Thus, while allowing students to reflect on the value of cognitively effortful tasks may be beneficial, the explicit appraisal of this value by others could, therefore, be even more effective to positively alter youth’s appraisals of such effort. In summary, providing cognitively demanding tasks that match students’ interests, along with explicitly highlighting its value, could lead to an increased perceived value of such tasks. More importantly, enhancing the perceived value of intellectually demanding tasks holds the potential to facilitate the development of a favorable NFC, as individuals are inclined to pursue activities that resonate with their values and avoid those that do not (Schunk and Usher 2019). Furthermore, increased task value has been suggested to positively influence engagement in and enjoyment of learning activities, particularly when accompanied by perceived control (i.e., self-efficacy) over the task (Forsblom et al. 2022; Peng and Cherng 2023; Shao et al. 2020). As such, enhancing the perceived value of intellectually demanding tasks may enhance NFC development.

#### 3.3.2. Appraisal of Coping Potential and Motivational Relevance

Enhancing appraisals of coping potential could also foster the development of a positive NFC. Aligning tasks with students’ interests not only enhances appraisals of value but also positively impacts appraisals of coping potential during intellectually demanding activities, leading to higher levels of engagement, enjoyment, and persistence, and thus counteracting the possible negative effects of these cognitive demands (Fulmer and Frijters 2011; Fulmer et al. 2015; Milyavskaya et al. 2021). The appraisal of coping potential can be further enhanced through the explicit appraisal or positive reinforcement of cognitive effort exertion. Studies have demonstrated that explicit verbal appraisals not only increase enjoyment and persistence but also enhance self-efficacy with regard to challenging cognitive tasks (Droe 2012; Haimovitz and Henderlong Corpus 2011; Zarrinabadi and Rahimi 2022). For example, Zarrinabadi and Rahimi (2022) found that university students who were praised for their effort had more positive beliefs regarding their writing abilities compared to those praised for their intelligence or those who received no praise. Thus, through increasing the appraisal of coping potential (i.e., self-efficacy), explicit effort praise could be a potent strategy to promote NFC development.

Explicit effort praise not only has the potential to enhance the appraisal of coping potential but could also influence the appraisal of the motivational relevance of cognitively demanding tasks. According to the Secondary Reward Theory (Eisenberger 1992), the aversiveness of exerting (cognitive) effort can be reduced by pairing this effort with a reinforcer, such as praise, recognition, or other forms of positive reinforcement. During this process, the experience of effort may acquire secondary reward properties, making the act of engaging in cognitive effort intrinsically rewarding and consequently leading to increased approach behaviors toward situations allowing or requiring cognitive effort (i.e., the behavioral adaptation of NFC). This phenomenon was recently empirically validated in a student sample (Clay et al. 2022). Thus, rewarding cognitive effort through explicit praise has the potential to enhance the intrinsic reward of cognitive effort, promoting greater approach behavior toward cognitively demanding situations and thereby fostering positive NFC development.

#### 3.3.3. Appraisal of Enjoyability

Lastly, it could be beneficial for NFC development if the appraisal of the enjoyability of cognitive effort exertion is enhanced. Although enjoyability is not typically emphasized as a traditional appraisal dimension in the aforementioned theories, it may play an important role in the development of one’s NFC. Individuals with high levels of NFC seek out and engage in intellectually demanding activities because they *enjoy* them. Therefore, if the environment could increase the perceived enjoyment of these kinds of activities for youth, this could potentially foster their NFC development. One way to achieve this is through emotional contagion, or the tendency to imitate another individual’s emotional state, and thus experiencing and displaying the same emotion (Sonnby-Borgström and Jönsson 2004). Empirical research has shown that teacher enjoyment is indeed linked to enjoyment within the classroom, even after accounting for enjoyment in previous school years, and that this relationship is mediated by teacher enthusiasm (Frenzel et al. 2009). Since teacher enjoyment represents a more internal experience, which can be hard for students to pick up, it has been suggested that teacher enjoyment can be transferred to the students through teacher enthusiasm (Frenzel et al. 2009). Teacher enthusiasm encompasses a teaching style that manifests through observable behaviors, such as dynamic gestures, facial expressions, varied voice intonations, and the frequent use of humor (OECD 2019). If teachers or other important social actors could make it evident to young individuals that they enjoy engaging in cognitively effortful activities through the enthusiasm with which they approach and present such activities, these feelings of enjoyment might actually “spread” to the students. As a result, this could foster a greater appreciation for intellectual challenges in general, increasing the likelihood of students engaging in such activities and ultimately enhancing the development of NFC.

### 3.4. Modeling

Observational learning refers to the process of acquiring knowledge by observing the actions of others (i.e., models), either directly or indirectly (Bandura 1977, 1997). Role models are often viewed as experts from whom individuals can learn, such as parents or teachers (Bandura 1997). Observational learning has been extensively investigated since its introduction by Bandura (1977), and it has been empirically validated that individuals can assimilate skills, beliefs, emotions, strategies, or attitudes by observing others within their social environment (e.g., Craig et al. 2009; Jacobs and Eccles 2000; Ryan 2019).

With regard to the model of NFC development, these “expert” models possess great potential to enhance both learned cognitive skills and self-regulatory processes, two key components of the adaptive triangle of the model. For example, it has been demonstrated that consistent exposure to expert models successfully solving problems while articulating their cognitive processes enhances students’ problem-solving skills (Craig et al. 2009; Van Gog and Rummel 2010). Additionally, studies indicate that teachers and parents can positively influence self-efficacy beliefs and motivation in educational settings (e.g., Groenendijk et al. 2013; Phan and Ngu 2016; Thevenin et al. 2016). Therefore, parents, teachers, and other role models can play a pivotal role in fostering NFC by modeling behaviors and attitudes associated with high NFC. This can be achieved by actively engaging in and persisting with challenging cognitive activities, thereby demonstrating the rewarding and enjoyable aspects of such tasks. Moreover, by openly acknowledging and persevering through their own mistakes, they can instill students with a resilient and positive mindset when approaching intellectually demanding activities.

In addition to parents and teachers, peers and siblings have also been recognized as influential role models in shaping the development of self-efficacy in young individuals (Murphy 2015; Schunk and Zimmerman 2006). Since peers are perceived as more like oneself compared to parents or teachers, observing a peer’s success can provide valuable insights into one’s own potential to achieve similar outcomes. This can positively impact self-efficacy beliefs, thereby motivating individuals to attempt similar tasks themselves (Schunk 2003). Furthermore, peer models may also offer insights into behaviors or attitudes leading to favorable or unfavorable outcomes, prompting individuals to emulate behaviors associated with positive results (Gibson 2003). Accordingly, observing that peers are praised for engaging in cognitive effortful activities (i.e., explicit appraisal) not only boosts their own confidence but also encourages other learners to participate in similar activities that lead to such positive outcomes (i.e., being praised; Muir 2018). Consequently, the deployment of peer models not only has the potential to influence the self-regulatory processes of the NFC development model but could also positively affect the frequency with which students invest cognitive effort (i.e., behavioral adaptation of NFC). As increased cognitive effort is crucial for enhancing cognitive skills (Haith and Krakauer 2018), this approach will further reinforce the adaptive triangle within the model and ultimately promote NFC development.

### 3.5. Supporting Success Experiences

As previously noted, one approach to cultivate more success experiences is to provide optimally challenging tasks, where task demands align with or slightly exceed individual capacities (Shernoff et al. 2003). Nonetheless, the design of the learning environment can encompass various additional strategies to increase the likelihood of encountering success in intellectually demanding tasks. For instance, establishing a structured learning environment, by example through setting clear expectations and providing step-by-step guidance on how to complete them, is a well-supported method for increasing learning achievements (Cheon et al. 2019; Vansteenkiste et al. 2012). Furthermore, it has been shown that differentiated instruction, an approach in which teachers tailor their curriculum and instruction to optimize learning for all students, positively impacts student performance and achievement (Alsalhi et al. 2021; Muthomi and Mbugua 2014; Tomlinson 2017). As such, teachers can differentiate on different key elements in the classroom based on students’ interests and learning profiles. Lastly, offering constructive feedback before, during, and after these tasks prompts students to adapt their approaches and behaviors to meet expectations in the future (Aelterman et al. 2014; Aelterman et al. 2019; Jang et al. 2010). Constructive feedback, aimed at providing useful guidance or suggestions to improve performance, skills, or behavior, focuses on specific actions rather than personal traits and is most effective when given shortly after the behavior it addresses; its main goal is to empower the student by highlighting areas for improvement and offering actionable advice, often including both positive reinforcement and suggestions for enhancement (Bee and Bee 1998; Ovando 1994). Providing students with constructive feedback stands as one of the most impactful strategies to improve self-efficacy beliefs and motivation towards and value of cognitive activities (Aslam et al. 2021). Ultimately, these factors contribute to students’ attainment of their learning objectives and success experiences (Hattie 2009; Kamardeen 2013).

In conclusion, through aligning task demands with individual capacities (i.e., optimal challenge), establishing a structured learning environment, providing differentiated instructions, and offering timely constructive feedback, teachers and parents can elevate the probability of success experiences. This, in turn, enhances students’ self-efficacy beliefs and positive attitudes towards challenging cognitive tasks—two critical elements of the self-regulatory dimension of NFC. Furthermore, an environment that promotes success is also the ideal foundation for improving cognitive skills. Therefore, prioritizing the cultivation of success experiences could stand as a pivotal approach in nurturing NFC positive development.

## 4. Discussion

The main purpose of the present article was to elucidate possible key factors contributing to the development of NFC, defined as an individual’s tendency to seek out, engage in, and enjoy cognitive effortful tasks (Cacioppo and Petty 1982). Recent longitudinal studies, albeit limited, have found great interindividual variability in students’ developmental trajectories of NFC, suggesting that NFC is malleable, particularly among younger individuals (Bergold et al. 2022; Bergold and Steinmayr 2023; Bruinsma and Crutzen 2018; Preckel 2014). This implies that NFC can be developed. However, most research has focused on the predictive capacity of NFC itself, with limited attention given to the determinants influencing its development. The available studies on the development of NFC suggest a general mean-level decline from childhood to mid-adolescence, and a slight increase from mid-adolescence onward. Given its association with numerous positive outcomes, finding avenues to fostering NFC development in youth seems crucial. To address this gap, we employed an emergent trait theory—the CATT (Matthews 2017)—to propose a developmental model of NFC. Drawing from the existing literature on NFC, we outlined several core components of the model, including neurobiological and basic information-processing elements, intellectual abilities, learned cognitive skills, and self-regulatory processes. More importantly, we illustrated how they may interact, potentially driving cognitive effort investment over time. Finally, we suggested several avenues for cultivating NFC development and elaborated on their mechanisms using the proposed model. These included influencing the appraisal of cognitive effortful activities, modeling NFC-related behaviors, and shaping an environment that fosters success experiences. We also emphasized that establishing a supportive and optimally intellectually stimulating environment could serve as prerequisites for fostering positive NFC development, alongside the aforementioned strategies. However, the listed strategies are just propositions and are not meant to be exhaustive. Further investigation is needed to empirically validate the effectiveness of the suggested strategies and to explore additional methods for promoting NFC.

Nevertheless, several important considerations should be made. First, it is crucial to distinguish between NFC at the trait- and the state-level to fully comprehend how NFC development can be positively influenced. While traits describe people in general terms, such as someone who generally enjoys exerting cognitive effort, states explain specific behaviors in particular situations and can be seen as deviations from one’s typical developmental trend (e.g., DeYoung 2015; Hecht et al. 2019). The recent literature on personality development has consistently proposed that personality change occurs as a result of daily experiences of trait deviations (i.e., states) that accumulate over time (Wrzus and Roberts 2017). For example, consider an individual who generally exhibits low trait NFC. In a specific moment and situation, such as during an optimally challenging activity, this person might display heightened levels of enjoyment and engagement in cognitive activities beyond what would typically be expected based on their trait NFC score, indicating a high state NFC. This temporary deviation from their trait level NFC can prompt the individual to engage in deeper and more intensive thinking, thereby fostering their cognitive skills. Consequently, this investment in cognitive activities is likely to enhance their performance on similar tasks in the future, making these tasks more enjoyable. Only if this sequence of events recurs frequently and proves advantageous in addressing environmental challenges, lasting personality change can be anticipated (Wrzus and Roberts 2017). Thus, implementing the aforementioned strategies will probably not lead to enduring changes in one’s tendency to enjoy and engage in cognitive activities when only used a few times. Rather, consistent and repeated positive experiences with cognitive activities seem necessary for fostering enduring changes in NFC. By continually creating opportunities for individuals to find cognitive activities rewarding and enjoyable, it may be possible to promote a stable and lasting increase in their NFC, thereby enhancing their overall cognitive engagement and development.

Second, within this article, we primarily focused on how the environment can foster positive NFC development. However, this emphasis does not imply that contextual factors alone determine NFC development; individual factors likely also play a crucial role in its progression. According to the transactional theory of development, developmental outcomes arise from continuous reciprocal influences between the individual and their context, including parents, teachers, and peers (Sameroff 2009). Therefore, the environments in which individuals find themselves are not entirely independent of these individuals; rather, they are shaped by and interact with the individual’s characteristics, behaviors, and choices. For example, individuals actively select and evoke responses from their environments based on their preferences, interests, and developmental needs (Sameroff 2009). Those who find enjoyment in intellectually challenging activities may actively seek intellectually stimulating environments that align with their cognitive interests. Moreover, their enthusiasm and motivation for such tasks may also influence how others within the environment respond to them, thereby further shaping their intellectual development. Thus, while our focus has been on the potential influence of the environment on NFC development, we do not disavow the important role individuals play in shaping their own development through these interactions with their environment. However, we assume that if the environment purposefully and consistently applies the proposed strategies while providing a safe learning environment and sufficient cognitive stimulation, there is potential for the environment to positively influence the NFC development of youth.

Third, the strategies we proposed to foster NFC development primarily focus on enhancing the self-regulatory processes, learned cognitive skills, and behavioral adaptations within our model. Given that neural and basic information-processing mechanisms are generally considered less malleable than the aforementioned elements (Matthews 2017), we only addressed them briefly when introducing our model and did not explore them in depth. Nonetheless, we recognize their importance, as they are likely to exert a significant influence on NFC development. Although our strategies may not directly target these mechanisms, their impact is probably also fundamental to the overall developmental process of NFC. Investigating these neural and information-processing mechanisms in more detail is a task for future research to further elucidate their role in NFC development.

Fourth, it is apparent that the proposed avenues to foster positive NFC development mainly target youth. Considering the evidence suggesting great malleability of NFC in younger individuals, it becomes evident that creating conducive contexts for NFC development holds significant importance among younger populations, such as children and adolescents. Hence, the proposed strategies primarily target the home and school context, as we expect these younger individuals to derive greater benefits from an NFC promoting environment. However, while prioritizing the shaping of environments for younger populations is crucial, it is imperative to recognize that NFC development remains open to influence across the lifespan. By cultivating environments supportive of NFC development, individuals of all ages can continue to enhance their NFC, with possible benefits for their personal growth and well-being.

Finally, it is essential to emphasize that while the findings from existing longitudinal studies suggest that NFC is malleable, there are currently no studies that definitively demonstrate this through experimental evidence. Given NFC’s relevance across a variety of contexts, conducting intervention studies specifically designed to influence NFC should be a research priority in order to fill this critical gap in the psychological literature and provide conclusive proof of its malleability. Our paper seeks to offer a theoretical framework outlining the interacting factors that could shape NFC development over time, which future research should investigate, particularly in the context of intervention-based studies. Proving the malleability of NFC would not only advance theoretical understanding but also open new avenues for enhancing cognitive engagement and motivation in educational, professional, and everyday settings. Ultimately, this endeavor could better equip individuals to thrive in an increasingly complex and cognitively demanding world.

## Data Availability

Not applicable.

## Appendix A

**Table A1:** Investment traits closely related to NFC.

Construct	Definition	Example Item(s)	Similarities	Differences
Need for Cognition (NFC)	An individuals’ tendency to seek out, engage in, and enjoy thinking (Cacioppo and Petty 1982).	“Thinking is fun for me”; “I like to work on problems that require a lot of thinking” (Preckel and Strobel 2017).		
Typical Intellectual Engagement (TIE)	An individuals’ aversion or attraction to intellectually demanding activities (Goff and Ackerman 1992).	“I prefer my life to be filled with puzzles I must solve” (Goff and Ackerman 1992, p. 540).	Both hold significant predictive power regarding the effort individuals typically invest in everyday situations (e.g., Cacioppo et al. 1996; Furnham et al. 2009; Strobel et al. 2018).	TIE focuses on the extent of intellectual effort typically exerted by an individual, regardless of their motivation to do so, while NFC is more concerned with the positive feelings associated with cognitive effortful activities.
Epistemic Curiosity (EC)	A hunger for knowledge that drives individuals to acquire knowledge about the world, bridge information gaps, and resolve intellectual challenges (Berlyne 1978; Litman 2008).	“I enjoy learning something new and I like to find out more”; “I enjoy learning about subjects which are unfamiliar” (Litman and Spielberger 2003, p. 82).	Both refer to a cognitive motivation to partake in intellectually challenging endeavors.	EC, being more deficit-driven, revolves around bridging knowledge gaps, and is fostered by feelings of uncertainty or “not-knowing” (Berlyne 1978; Lamnina and Chase 2019; Litman 2005). NFC does not prioritize bridging knowledge gaps but centers around the enjoyment derived from engaging in cognitive activities (Cacioppo et al. 1996).
Openness to Ideas (OI)	One’s inclination to embrace and consider new concepts, thoughts, and perspectives, which can be reflected in an active pursuit of intellectual activities for their intrinsic value (Costa and McCrae 1992; McCrae and Sutin 2009).	“Has high degree of intellectual capacity”; “Concerned with philosophical problems” (Costa and McCrae 1995, p. 31).	Both relate to the pursuit of and engagement in intellectual activities, and this being not for extrinsic reasons but for the intrinsic value of the task for hand (Fleischhauer et al. 2009).	While OI demonstrates stronger predictive power for constructs associated with novelty and the pursuit of variety, NFC exhibits a more goal-oriented and cognitive dimension, being more closely linked to a general inclination to actively invest cognitive resources irrespective of context (Fleischhauer et al. 2009).

## Appendix B

**Table A2:** Variables associated with higher NFC levels.

Dimension	Empirical Evidence
Neural correlates	Larger gray-matter volume in brain regions involved in motivational and visuospatial processes (Liu and Nesbit 2023; Tolomeo et al. 2023). Greater brain flexibility in various brain areas and networks (He et al. 2019).
Basic information-processing	Increased involuntary attention allocation to task-relevant stimuli as indicated by electrocortical indices (Enge et al. 2008; 2011; Strobel et al. 2015). More efficient neuronal information-processing (Mussel et al. 2016). Lower reward when high cognitive effort is avoided (Gheza et al. 2023).
Intellectual abilities	Stronger intellectual abilities (e.g., Bergold and Steinmayr 2023; Lavrijsen et al. 2021; Von Stumm and Ackerman 2013).
Learned cognitive skills	Stronger problem-solving skills (Coutinho et al. 2005; Nair and Ramnarayan 2000; Rudolph et al. 2018). Improved task focus (Levin et al. 2000; Li and Browne 2006; Srivastava et al. 2010). More frequent use of strategies that enhance explicit information-processing (e.g., Mokhtari et al. 2013) and deeper learning (Cazan and Indreica 2014).
Self-regulatory processes	Greater enjoyment during and positive attitude toward intellectually demanding tasks (e.g., Li and Browne 2006; Weissgerber et al. 2018). Preference for complex over simple problems (See et al. 2009; Therriault et al. 2014; Zerna et al. 2023). Enhanced self-efficacy beliefs in learning or academic contexts (e.g., Elias and Loomis 2002; Naderi et al. 2018).
Behavioral adaptation	More willing to exert cognitive effort (e.g., Kramer et al. 2021). More frequent cognitive effort exertion (e.g., Cazan and Indreica 2014; Therriault et al. 2014). Greater engagement in and persistence during effortful cognitive activities (Dickhäuser et al. 2009; Fleischhauer et al. 2015; Lavrijsen et al. 2023).

## Appendix C

**Table A3:** Overview of the suggested strategies to promote NFC development.

Strategy	Definition	Strategies
Safe learning environment	An educational setting characterized by supportive and responsive interpersonal relationships with significant others, which provides a foundation of care, acceptance, and support.	Providing a safe haven for students to seek comfort during the distress that comes with cognitively demanding tasks.
Optimal challenge	An educational setting where cognitive tasks and activities are designed to align with or slightly exceed an individual’s current capabilities.	Offering tasks and activities to students that are sufficiently challenging to stimulate interest and achievement without being overwhelming.
Appraisal of cognitive activities	Emotional responses to cognitively demanding tasks are shaped by evaluating such tasks across various appraisal dimensions, resulting in differences in emotional and behavioral reactions depending on how such tasks are perceived.	Influencing how students appraise cognitive tasks through offering tasks that align with their interests, clearly emphasizing the value of these tasks, providing explicit praise for their efforts, and having significant others approach these tasks with enthusiasm.
Modeling	The process of acquiring skills, beliefs, attitudes, or behaviors related to higher levels of NFC by observing the actions and attitudes of others, such as significant others or peers.	Exposing students to “expert models”, like teachers or parents, who actively engage in challenging cognitive activities while articulating their cognitive processes and demonstrating their value and enjoyment while also openly acknowledging and learning from their own mistakes. Exposing students to “peer models” who frequently succeed in cognitively demanding tasks and receive praise for their efforts.
Supporting success experiences	An educational setting that enhances the likelihood of success experiences for students.	Providing tasks that align with individual capacities, establishing a structured learning environment, offering differentiated instruction tailored to students’ learning profiles and interests, and delivering timely constructive feedback.
